# Historical Pandemic and Contemporary Influenza A Viruses Reveal PB2 M631L as a Convergent Adaptation to Human ANP32

**DOI:** 10.3390/microorganisms14040859

**Published:** 2026-04-11

**Authors:** Matthias Budt, Irina Barac, Jessica Kohs, Tim Krischuns, Nadia Naffakh, Thorsten Wolff

**Affiliations:** 1 Unit 17 Influenza and other Respiratory Viruses, Robert Koch Institute, 13353 Berlin, Germany; budtm@rki.de (M.B.); irinabarac8@gmail.com (I.B.); jessi.kohs@gmx.de (J.K.); 2 Institut Pasteur, Université Paris Cité, CNRS UMR 3569, RNA Biology of Influenza Virus, 75015 Paris, France; tim.krischuns@bioquant.uni-heidelberg.de (T.K.); nadia.naffakh@pasteur.fr (N.N.)

**Keywords:** influenza A virus, 1918 Spanish influenza, pandemic, H5N1, polymerase, ANP32, PB2 M631L

## Abstract

Understanding the genetic changes that allow avian influenza A viruses (IAVs) to switch their natural hosts and establish productive infection in humans is important for pandemic risk assessment. Adaptations in the IAV polymerase are required to overcome species-specific restrictions imposed by host ANP32 proteins. Notably, avian virus polymerase is generally only poorly supported by human ANP32 proteins due to species-specific differences. Consequently, efficient polymerase adaptation to the binding interface of human ANP32 requires distinct amino acid changes, such as PB2 E627K. A separate adaptation, PB2 M631L, has recently been reported in mammalian-adapted IAV; however, its functional role across divergent viral lineages and its relationship to host ANP32-dependent adaptation remain incompletely defined. Here, we examine PB2 M631L in the polymerases of a 1918 pandemic strain, a recombinant contemporary H1N1pdm09, and a recent clade 2.3.4.4b H5N1 virus. Using polymerase activity and protein-interaction assays, we show that PB2 M631L enhances polymerase activity and ANP32 binding in human—but not avian—contexts, and that this effect is conserved across multiple viral backgrounds. In H1N1pdm09, PB2 M631L also increased virus replication in mammalian cells. These findings indicate that PB2 M631L contributes to enhanced polymerase compatibility with human ANP32 proteins and are consistent with a role in adaptation across multiple influenza virus lineages. Our results highlight how analysis of historical pandemic strains can inform risk assessment for future emerging viruses.

## 1. Introduction

Influenza A viruses (IAVs) pose a major public health threat, not only for causing annual epidemics with substantial morbidity and mortality, but also for their potential to spark pandemics with widespread disease and severe outcomes. IAVs are classified into subtypes based on the surface proteins hemagglutinin (HA) and neuraminidase (NA), of which 19 and 11 variants exist, respectively. Seasonal epidemics arise from the continual evolution of circulating human H3N2 and H1N1 strains, whereas pandemics occur when a novel virus emerges to which the population lacks immunity. Wild birds are the natural IAV reservoir, and the pandemics of 1918, 1957 (“Asian Flu”), and 1968 (“Hong Kong Flu”) resulted from avian viruses transmitted to humans after reassortment. In contrast, the 2009 H1N1 pandemic originated from swine [1,2]. The 1918 Spanish Flu was the most severe, causing an estimated 50 million deaths and infecting roughly one third of the global population [3]. Despite its scale, only limited viral sequences from 1918 are available [4], and functional studies focus largely on the reference strain A/Brevig Mission/1/1918 (H1N1) [5]. Molecular studies identified the viral receptor binding protein hemagglutinin (HA) and the viral RNA polymerase as the main virulence and pathogenicity determinants [6,7,8,9]. Recent advances in sample preparation and sequencing have uncovered additional 1918 virus genomes, expanding knowledge of its evolution and diversity [10,11,12].

While the pandemics of 1957 and 1968 were caused by H2N2 and H3N2 viruses, the 2009 pandemic virus belonged to the H1N1 subtype (H1N1pdm09) but was distantly related to the 1918 strain. First detected in Mexico, it resulted from complex reassortments among swine, human, and avian IAV, with its polymerase genes derived from avian (PA and PB2) and human (PB1) lineages that entered swine around 1998 [13,14].

Highly pathogenic avian influenza H5N1 of the goose/Guangdong lineage emerged in 1996 in poultry and has since caused widespread outbreaks in poultry and wild birds across nearly all continents [15]. Its circulation changed markedly after 2021 with the emergence of clade 2.3.4.4.b strains, which infect a broad host range, including wild birds, poultry, and occasionally mammals, including humans [16]. An unprecedented outcome is the ongoing H5N1 outbreak in U.S. dairy cattle since early 2024, with 1084 affected herds in 19 states as of 19 January 2026 [17]. The virus replicates to high titres in the udder, is shed in milk, and spreads between cows during milking [18]. Notably, 71 human H5N1 clade 2.3.4.4.b infections have been reported in the U.S., mostly linked to poultry or cattle exposure [19]. This situation carries major public health and economic implications and has intensified efforts to understand and monitor viral traits that could enable a future pandemic.

To achieve sustained human transmission, zoonotic IAV must acquire mutations that allow efficient use of human host factors for replication and shedding. The viral ribonucleoprotein complex, comprising viral RNA with polymerase subunits PB1, PB2, PA, and nucleoprotein NP [20], drives viral RNA transcription and replication. For optimal activity, the polymerase interacts with host proteins such as importins [21] and cellular RNA polymerase II to enable cap snatching of its transcripts for priming viral mRNA synthesis [22,23,24]. A key group of host cofactors is the ANP32 phosphoproteins: binding of human ANP32A or ANP32B promotes formation of an asymmetric polymerase dimer essential for genome replication [25,26]. Importantly, ANP32 proteins vary across species; only avian ANP32A supports high replication efficiency and contains a 33-amino acid insertion that is absent from mammalian homologs [27]. Thus, avian viruses require adaptive polymerase mutations to use the shorter mammalian ANP32A/B proteins. The best-characterized adaptation is PB2 E627K that enhances electrostatic interactions with mammalian ANP32 [28]. Structural studies suggest that the E627K exchange results in a positively charged surface that could better accommodate electrostatic interactions with the C-terminal acidic tail of mammalian ANP32 [24,26]. Additional spillover-associated PB2 mutations cluster in the same region. Notably, we recently identified a PB2 M631L polymorphism in a 1918 pandemic genome from Germany, though its relevance remains unknown [10]. The 2009 H1N1pdm PB2 retained avian type 627E but carried the GQ590/591SR and T271A adaptations [29]. In H5N1 clade 2.3.4.4.b, several ANP32-related mutations have appeared, including E627K in a human case, M631L in cattle isolates, and Q591K and D701N in marine mammals [30,31,32].

PB2 M631L has been reported previously in mammalian-adapted IAV, including mouse-adapted strains and recent clade 2.3.4.4b H5N1 viruses. In these systems, the mutation enhances polymerase activity and pathogenicity in vivo [33,34,35]. However, how this mutation functions across distinct viral backgrounds and historical pandemics, and how it relates to host ANP32 specificity, has not been systematically examined in a comparative functional framework spanning multiple IAV lineages, nor has its relationship to ANP32-dependent host adaptation been clearly defined.

We therefore investigated the impact of PB2 M631L on polymerase function across virus strains spanning more than a century, including the 1918 and 2009 pandemic viruses and current H5N1. We show that PB2 M631L selectively enhances polymerase activity and ANP32 binding in human, but not avian, contexts. Mechanistically, PB2 M631L strengthens polymerase binding to human ANP32A and, to a lesser extent, ANP32B. This comparative approach allows us to place PB2 M631L within a broader evolutionary and host-factor context and to assess whether this mutation contributes to polymerase adaptation that enhances compatibility with human ANP32 proteins across divergent IAV lineages. Our results highlight how analysis of historical pandemic viruses can inform the interpretation of adaptive mutations observed in emerging influenza strains.

## 2. Materials and Methods

### 2.1. Cells

Human HEK-293T (human embryonic kidney) and MDCK-II (Madin–Darby canine kidney) cells were cultured in Dulbecco’s modified Eagle medium (DMEM) or Eagle’s minimum essential medium (MEM), respectively, supplemented with 10% fetal bovine serum and (FBS; Biochrom, Berlin, Germany), 2 mM L-glutamine (Roth, Karlsruhe, Germany), 100 U/mL penicillin and 100 U/mL streptomycin. HEK-293T cells lacking expression of ANP32A and ANP32B were described [24]. Calu-3 cells were propagated in DMEM with 15% FBS, glutamine, pen/strep as above, 1× nonessential amino acids and 1 mM sodium pyruvate. All the cells were grown at 37 °C in a humidified atmosphere with 5% CO2 and were regularly screened for mycoplasma.

### 2.2. Plasmids, Mutagenesis and Transfection

The pCAGGS plasmids for expression of IAV polymerase and nucleoprotein CDS derived from German 1918 (MU-162) or of strain A/Brevig Mission/1/1918 (BM) and generation of mutants were described [10]. The viral cDNAs corresponding to consensus sequences of H5N1 cattle were commercially synthesized according to sequences published elsewhere [36]. The sequences are shown in Supplemental Figure S2. Mutagenesis of polymerase segments and cloning into pCAGGS were performed as described [10,37]. Briefly, polymerase protein coding sequences were PCR-amplified with primers containing BsaI sites using Phusion polymerase according to the manufacturer’s instructions. Purified PCR products were then cloned into pCAGGS-Esp-blue in a one-pot cloning reaction with simultaneous restriction and ligation. Constructs were controlled by Sanger sequencing of the complete inserts. The primers used for cloning and mutagenesis are listed in Table S1. PB2 sequences of the viruses used in this study are shown in Figure S1.

### 2.3. Polymerase Activity Assay

Trypsinized HEK-293T-ANP32-A/B-double-ko cell cultures were transiently transfected in suspension in 12-well plates using lipofectamine 2000 (1.5 µL per µg DNA; Invitrogen, part of ThermoFisher, Dreieich, Germany) with 50 ng of each pCAGGS-pol plasmid; 100 ng pCAGGS-NP plasmid; 125 ng of pPolI-NSLuc, expressing an IAV-like RNA encoding a firefly luciferase and 30 ng of pcDNA3.1-ANP32 expression plasmid [24]. Transfection was normalized by the constitutively expressed Renilla luciferase encoded on plasmid pTK-RL (10 ng; Promega, Madison, WI, USA). The total DNA amount was equalized in every sample with pCAGGS. At 24 h post transfection, luciferase activity was measured with the Dual-Luciferase^®^ Reporter Assays System (Promega, Madison, WI, USA) on a Tristar LB 941 luminometer (Berthold Technologies, Bad Wildbad, Germany) according to the manufacturer’s instructions. For transfection normalization, relative light units (RLU) of untransfected cells were subtracted from the samples separately for firefly and Renilla luciferases, and then the relative polymerase activity was calculated by dividing firefly by Renilla values.

### 2.4. Protein–Protein Complementation Assay

The G. princeps split-luciferase-based protein–protein complementation assays were performed as described [38] with minor modifications. The PB1 ORFs were tagged C-terminally with the G. princeps G2 fragment by exchanging PB1 in the WSN pCI-PB1-G2 plasmid. ANP32 plasmids tagged C-terminally with the G. princeps G1 fragment were described [24]. Briefly, 4.5 × 10^4^ HEK293T cells were seeded per 96 well and transfected after 18 h with 100 ng of each polymerase or ANP32 expression plasmid using 2 µL polyethyleneimine (PEI, 2.5 g/L, Sigma-Aldrich, St. Louis, MO, USA) per µg DNA. At 24 h post transfection, luciferase activity was measured with the Renilla Luciferase Assay System (Promega, Madison, WI, USA). The cells were lysed in 50 µL of Renilla lysis buffer for 30 min and the Gaussia princeps luciferase enzymatic activity was measured on a Tristar LB941 luminometer (Berthold Technologies, Bad Wildbad, Germany) with 10 s integration time after injection of 50 µL of Renilla luciferase assay reagent. Mean relative light units (RLUs) of technical triplicates are represented after subtraction of values for untransfected cells. In every experiment, we used two negative controls by omitting either the PB1-G2-Luc or the ANP32-G1-Luc construct, respectively.

### 2.5. Immunoblotting

Equal amounts of cell lysates from luciferase assay replicates were pooled; denatured in reducing SDS-PAGE sample buffer for 5 min at 95 °C; separated on 8% SDS gels; subjected to immunoblotting with antibodies against PB2 (rabbit, ThermoFisher/Pierce, Dreieich, Germany, #PAS-32221, used at 1:2000 dilution), PB1 (rabbit, ThermoFisher/Pierce, Dreieich, Germany, #PAS34914, 1:1000), PA (rabbit, GeneTex, Freising, Germany, #125932, 1:1000), NP (mouse, Acris, Herford, Germany, #AM00929PU-N, 1:2000) and actin (mouse, Sigma-Aldrich, St. Louis, MO, USA, #A2228, 1:5000), respectively and detected with HRP-labeled anti-mouse (DAKO, Glostrup, Denmark, #P0260) or anti-rabbit antibodies (DAKO, Glostrup, Denmark, #P0217), respectively.

### 2.6. Generation of Recombinant Viruses and Analysis of Virus Replication

Recombinant viruses of A/Hamburg/04/2009 variants were generated using an eight-plasmid system based on pHW2000 by transfection of human HEK-293T cells, followed by passage on MDCK-II cells [39]. The cells were plated on 35 mm dishes precoated for 15 min with 40 µg/mL of poly-D-lysine (Sigma-Aldrich, St. Louis, MO, USA). After 18 h, the cells were transfected with eight pHW2000 plasmids, carrying one IAV gene segment each. A total of 0.5 µg of each plasmid was mixed in 100 µL Opti-MEM (ThermoFisher, Dreieich, Germany) and combined with 4 µL Lipofectamine 2000 in 100 µL Opti-MEM. The transfection mixture was incubated for 15 min at room temperature, and then added to the cells for 6 h. The cells were washed and incubated in FCS-free medium with 0.25 µg/mL of TPCK-trypsin (Sigma-Aldrich, St. Louis, MO, USA). At 72 h post transfection, 500 µL of the cleared supernatant was passaged twice on MDCK-II cells in T25 flasks for 48 h each. The second passage supernatant was aliquoted and stored at −80 °C. Virus titres were determined by plaque assay. MDCK-II cells in 12-well plates were infected with 100 µL of serial dilutions of virus stocks for 45 min at room temperature. The cells were washed with PBS and incubated with semi-solid plaque medium containing 1.25% Avicel (RC-581, FMC Corporation, Philadelphia, PA, USA) at 37 °C for 48 h. The cells were washed, fixed in 2.5% formaldehyde and stained with crystal violet for 30 min. Plaques were counted in triplicate and stock titres calculated in pfu/mL. For analysis of virus replication, the MDCK-II or Calu-3 cells growing in 12-well plates were infected in triplicate at an MOI of 0.001 or 0.01, respectively, for 45 min. After washing in PBS, 1 mL of serum-free medium containing 1 µg/mL TPCK-trypsin was added and the cells were incubated at 37 °C. A total of 100 µL supernatant was removed at the indicated time points for plaque titration and replaced with fresh medium. For the plaque morphology analysis in Figure 4, incubation was done for 72 h at 33 °C, the condition that allowed the best simultaneous display of all the plaque phenotypes.

### 2.7. Virus Genome Sequencing

Sequencing of recombinant viruses was performed as described [40]. Briefly, the viral genome was amplified from RNA using a multi-segment one-step RT-PCR with Superscript III high-fidelity RT-PCR kit (Invitrogen, Carlsbad, CA, USA) according to the manufacturer’s instructions with universal primers. The RT-PCR amplification parameters were: 2 min at 55 °C; 60 min at 42 °C; and 2 min at 94 °C; followed by 5 cycles of 94 °C/30 s, 44 °C/30 s, and 68 °C/3.5 min; 26 cycles of 94 °C/30 s, 57 °C/30 s, and 68 °C/3.5 min, and a final extension for 10 min at 68 °C. Amplicons were visualized on a 1% agarose gel and purified with Agencourt AMPure XP beads (Beckman Coulter, Brea, CA, USA). The concentration of purified amplicons was quantified using the Qubit High Sensitivity dsDNA kit and a Qubit Fluorometer (Invitrogen, Carlsbad, CA, USA). The samples were sequenced on a NovaSeq 6000 platform (Illumina, San Diego, CA, USA).

### 2.8. Statistical Analysis and Software

**Figure 1:**
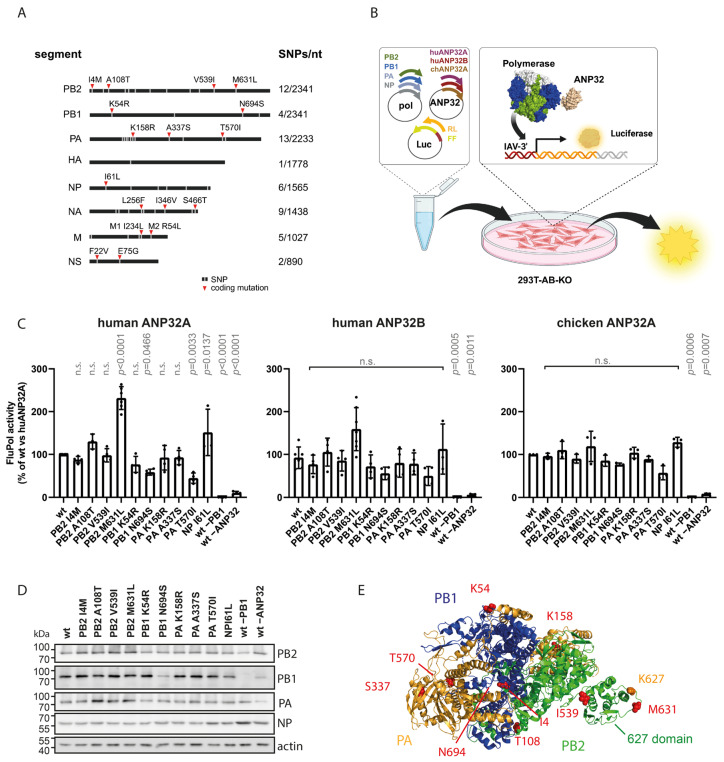
A 1918 influenza virus variant from Munich, Germany, carries the PB2 M631L polymorphism: (**A**) Polymorphisms in the recently identified German 1918 virus (MU-162, [10]) compared with the reference strain A/Brevig Mission/1/1918. Ten of 17 coding mutations map to polymerase complex subunits PB2, PB1, PA, and NP. (**B**) Principle of the ANP32-complementation mini-replicon assay: HEK-293T ANP32A/B double-knockout cells (293T-AB-KO) were transfected with expression plasmids for viral polymerase and NP, an ANP32 plasmid, a negative-sense firefly luciferase (FF) reporter under an influenza promoter, and a constitutive Renilla luciferase (RL) for normalization. Co-expression of viral polymerase and cellular ANP32 promotes expression of luciferase that is used to quantify polymerase activity. (**C**) Activity of Brevig Mission (BM) polymerase or BM variants carrying individual German 1918 amino acids was measured using human ANP32A, human ANP32B, or chicken ANP32A. The values are means ± SD, normalized to the activity of BM wt with huANP32A. Dots indicate *n* = 3–9 biological replicates with two technical replicates each. Statistical difference of mutants to wt was determined by nested ANOVA; *p*-values are indicated; n.s., not significant. (**D**) Polymerase protein levels were assessed by immunoblot. (**E**) In the IAV polymerase structure (PDB 6QNW [7]), variant residues are shown as red spheres. PB2 position 631 lies near the canonical adaptive residue 627 within the 627 domain. Created with BioRender.

**Figure 2:**
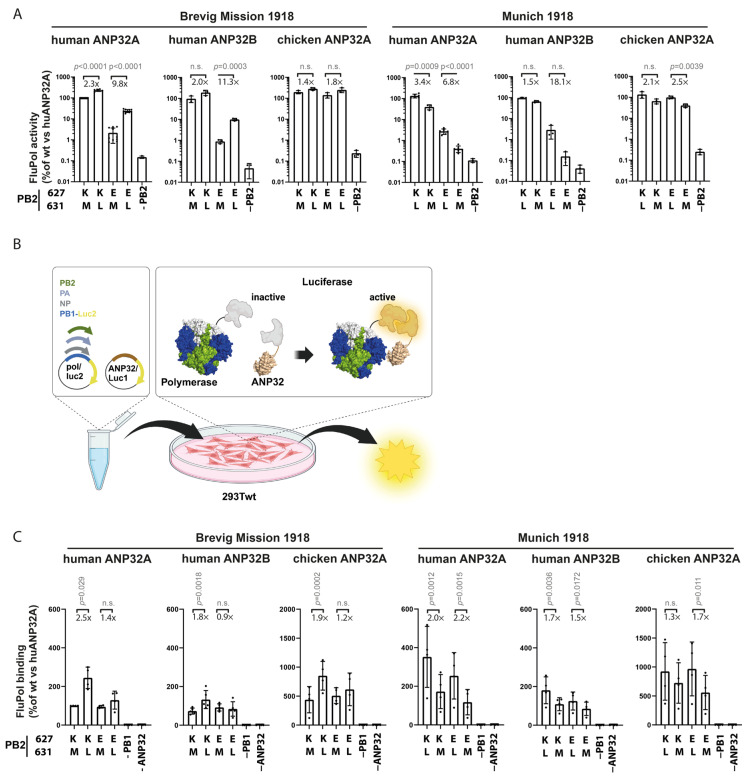
Polymerase activity of 1918 PB2 M631L variants in human ANP32 contexts independent of E627K: (**A**) Polymerase activity of Brevig Mission variants was assessed in 293T-AB-KO cells complemented with human ANP32A, human ANP32B, or chicken ANP32A. As the German 1918 polymerase contains PB2 631L, mutations were introduced reciprocally. Wild-type configurations are displayed in the first bar from the left for both polymerases. Values are means ± SD, normalized to the activity of BM wt polymerase with huANP32A. Dots represent *n* = 3–11 biological replicates with two technical replicates each. Numbers indicate fold changes of 631L vs. 631M variants. (**B**) Principle of the split luciferase FluPol binding assay. ANP32 and PB1 variants were fused to the N-terminal fragments of Gaussia luciferase. Interaction within the polymerase complex reconstitutes active luciferase. (**C**) Impact of PB2 M631L and E627K on polymerase binding to ANP32 proteins. Values are means ± SD normalized to BM wt binding to huANP32A. Dots represent *n* = 4 biological replicates with three technical replicates each. Significance was determined by nested two-tailed *t*-tests; *p*-values are indicated. n.s., not significant. Created with BioRender.

**Figure 3:**
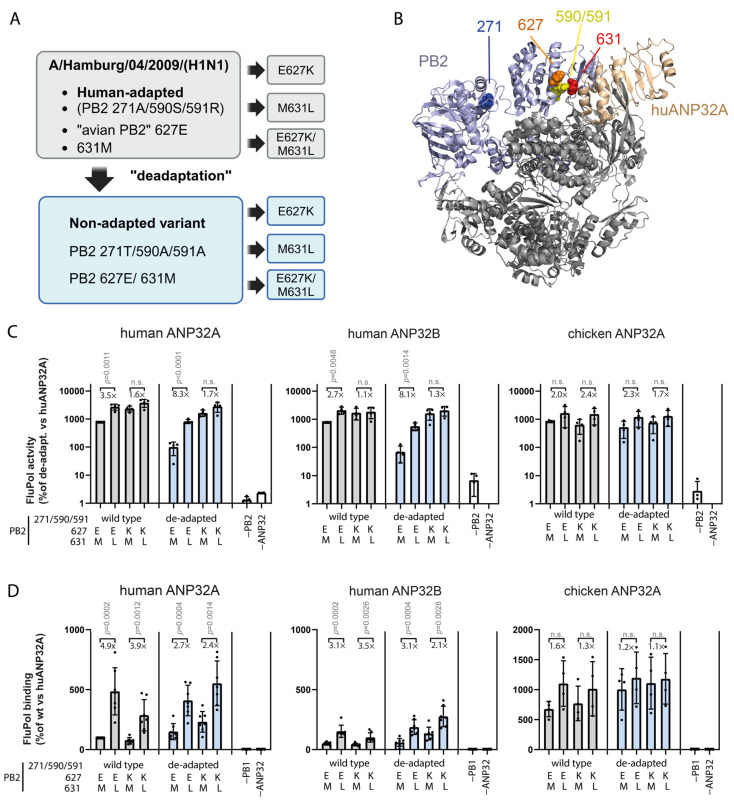
Different adaptive sites for the 2009 polymerase cluster at the ANP32A binding interface: (**A**) Human-adaptive PB2 residues 271A/590S/591R of A/Hamburg/04/2009 were reverted to the avian-like T271/A590/A591 variant. PB2 E627K and M631L mutations were then introduced individually or together. (**B**) Ribbon diagram of the structure of the IAV polymerase–huANP32A complex (PDB XYZ [8]) with adaptive residues highlighted as spheres. (**C**) Polymerase activity of A/Hamburg/04/2009 variants was tested with huANP32A, huANP32B, or chicken ANP32A. WT and de-adapted variants are shown in gray and light blue. Values are means ± SD normalized to the de-adapted variant with huANP32A (set to 100%). Fold changes indicate 631L vs. 631M. Dots represent *n* = 3–4 biological replicates with technical triplicates. (**D**) Polymerase–ANP32 binding measured by FluPol binding assay. Values are means ± SD normalized to wt binding to huANP32A. Dots indicate *n* = 4–7 independent biological replicates done in technical triplicates each. Fold changes indicate 631L vs. 631M. Significance was evaluated using a nested two-tailed *t*-test; *p*-values are indicated. n.s., not significant. Created with BioRender.

**Figure 4:**
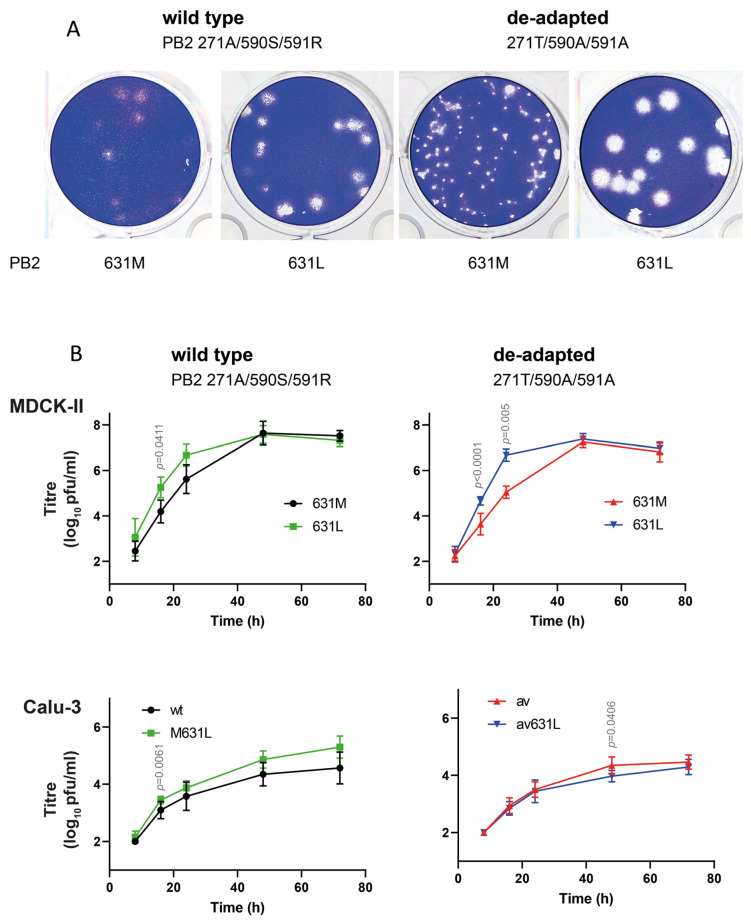
PB2 M631L enhances replication of 2009 pandemic-like viruses in mammalian cells. (**A**) Recombinant A/Hamburg/4/2009 viruses carrying the human-adapted or de-adapted PB2 signatures and either 631M or 631L were used to infect MDCK-II cells. After infection, cells were overlaid with Avicel containing 1 µg/mL TPCK-trypsin. Plaques were visualized after 3 days at 33 °C by crystal violet staining. (**B**) Canine MDCK-II cells were infected at M.O.I 0.01 and viral titres in supernatants quantified by plaque assay. The human bronchial epithelial cell line Calu-3 was infected at M.O.I. 0.1. Symbols represent *n* = 3–4 biological replicates with technical duplicates or triplicates. Values are means ± SD. Significance was determined using repeated-measures two-way ANOVA; *p*-values for significant differences are indicated.

**Figure 5:**
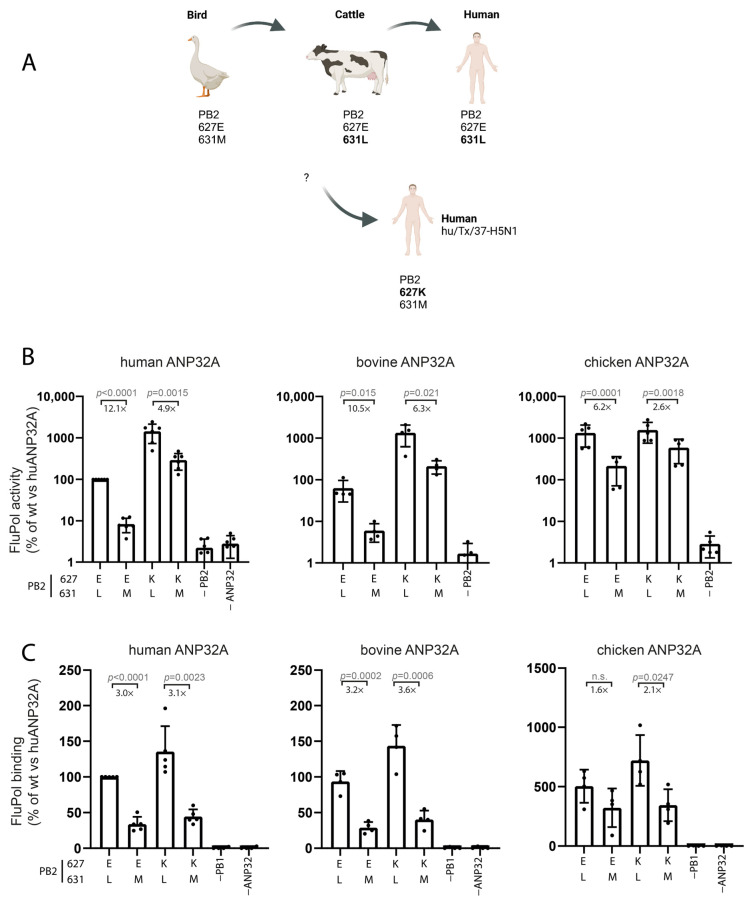
PB2 M631L and E627K independently enhance H5N1 cattle polymerase activity in human and bovine ANP32 contexts. (**A**) In the ongoing H5N1 clade 2.3.4.4b outbreak, some bird-derived viruses infecting humans contain PB2 E627K (e.g., A/Texas/37/2024), whereas cattle-associated viruses carry PB2 M631L. (**B**) Activity of H5N1 cattle polymerase variants encoding PB2 627E/627K and 631M/631L in all combinations, assessed by ANP32 complementation (see Figure 1B). Values are means ± SD of *n* = 4–6 biological replicates with technical duplicates each. Numbers reflect fold changes of 631L vs. 631M. (**C**) Polymerase–ANP32 binding measured in FluPol binding assays (Figure 2C). Values are means ± SD. Dots represent *n* = 3–4 biological replicates with technical triplicates each. Significance was evaluated using a nested two-tailed *t*-test; *p*-values are indicated. n.s., not significant. Created with BioRender.

The graphs and bars display mean values, with the error bars indicating standard deviations. Biological replicates refer to independent experiments performed on different days, whereas technical replicates represent repeated measurements within the same experiment. The numbers for each assay are given in the figure legends. Calculations were performed with GraphPad Prism, version 10.6.0. Normality of residuals was evaluated using the Shapiro–Wilk test. For assessment of the hypothesis that significant differences in polymerase activity among multiple variants might exist (Figure 1C), nested ANOVA with Dunnett’s correction for multiple comparisons was used to account for technical replicates within biological experiments. To test the hypothesis that mutations at PB2 position 631 significantly increase or decrease polymerase activity (Figure 2A, Figure 3C and Figure 5B) or ANP32 binding (Figure 2C, Figure 3D and Figure 5C) relative to the parental variant, nested two-tailed *t*-tests were performed to account for technical replicates within independent biological experiments. To evaluate the hypothesis that PB2 M631L might increase or decrease virus replication over time (Figure 4B), viral titers were log-transformed and assessed for normality using the Shapiro–Wilk test. Subsequently, data were analyzed by repeated-measures two-way ANOVA with Tukey’s post hoc correction for multiple comparisons. Primary data and statistical analysis are presented in Supplementary File S1. Images were generated with Biorender. Budt, M. (2025) https://BioRender.com/w76x488.

## 3. Results

### 3.1. PB2 M631L Enhances ANP32A-Dependent Polymerase Activity in a 1918 Variant

Since only a few viral sequences from the 1918 “Spanish Influenza” pandemic have been characterized, our understanding of how this avian-derived virus achieved efficient human replication and transmission is limited and requires further investigation. We recently identified a complete 1918 genome from a victim in Munich, Germany (MU-162), containing ten polymerase-complex polymorphisms relative to A/Brevig Mission/1/1918 (Figure 1A) [10]. We hypothesized that some of these substitutions contributed to human adaptation. To test this, we generated mutant plasmids allowing reconstitution of Brevig Mission polymerase with each MU-162 residue and assessed activity using a mini-replicon assay (Figure 1A). To dissect ANP32 dependence, we used 293T ANP32A/B double-knockout cells complemented with human ANP32A or ANP32B, or chicken ANP32A (Figure 1B). As expected, polymerase activity required ANP32 co-expression (Figure 1C).

Most MU-162 polymorphisms resulted in unchanged or reduced activity, consistent with earlier findings [10]. Notably, the variation PB2 M631L significantly increased polymerase activity in the presence of human ANP32A, and slightly heightened activity in conjunction with human ANP32B, although this effect was statistically not significant. In contrast, neither PB2 M631L nor any other mutant boosted activity when co-expressed with chicken ANP32A (Figure 1C). Chicken ANP32A generally supported higher polymerase activity, but in a similar manner for all the mutants. Comparable expression levels of most variant polymerase proteins were confirmed by immunoblotting (Figure 1D). One variant, PB1 N694S, showed lower expression levels, indicating a potential impact of this polymorphism on polymerase protein stability. Of note, prior analysis in 293T cells [10] had not revealed an impact of PB2 M631L on polymerase activity. These differences likely reflect the removal of endogenous ANP32 proteins and the controlled reconstitution of defined ANP32 variants. PB2 631 lies adjacent to the well-known adaptive residue 627 (Figure 1E), and M631L has been linked to mouse adaptation of H10N7 and enhanced avian virus replication, in ANP32A-mutant chickens and most recently in H5N1 spillover to cattle (Table 1). While PB2 627E dominates in avian polymerases, most human-adapted polymerases including the 1918 Brevig Mission and Munich variant strains contain 627K, which promotes adaptation to huANP32A/B. These observations suggested that M631L, alone or with 627K, may have enhanced replication of the 1918 virus in human cells, likely facilitated by increased polymerase activity.

To evaluate the role of PB2 M631L for viral polymerase activity, we engineered Brevig Mission and MU-162 polymerases encoding 631M or 631L combined with either 627E or 627K. Mini-replicon assays with ANP32A or ANP32B showed that 631L consistently increased activity relative to 631M. The effect was strongest in 627E backgrounds (Figure 2A). For instance, with huANP32A, Brevig Mission 627E/631L was 9.8-fold more active than 627E/631M, whereas the 627K/631L gain was only 2.3-fold. Similar results were observed with MU-162 and with huANP32B complementation. Thus, with ANP32A, Munich-162 with 627E/631L was 6.8-fold more active than the corresponding 627E/631M variant, while the 627K/631L variant was only 3.4-fold more active than the 627K/631M polymerase. In cells expressing huANP32B, the 627E/631L variants showed 11.3-fold or 18.1-fold higher activity compared to the 627E/631M variants for Brevig Mission and Munich-162, respectively. In contrast, the corresponding activity changes in the 627K/631M versus 627K/631L polymerases were not statistically significant. Chicken ANP32A supported similar activity across all the variants, with a maximum of 2.5-fold increase for 627E/631L compared to 627E/631M for Munich-162. In all the other settings with chANP32A, the effects of M631L were weaker and not significant.

We next tested whether enhanced activity correlated with improved ANP32 interaction using a previously established [24] split-luciferase assay where we fused the N-terminal and C-terminal parts of the G. princeps luciferase to ANP32 and polymerase PB1 proteins, respectively, and expressed them with the remaining polymerase components in 293T cells. The binding of polymerase to ANP32 then allows for luciferase reconstitution and activity (Figure 2B). In this assay, PB2 M631L increased the binding of both 1918 polymerases to huANP32A, with weaker effects for huANP32B and chicken ANP32A (Figure 2C). As reported previously, PB2 E627K did not increase this interaction [43].

Together, these data show that PB2 M631L enhances 1918 polymerase activity and binding to human ANP32A in the experimental systems used.

### 3.2. Impact of PB2 M631L on Polymerase Activity and Replication of 2009 Pandemic IAV

We next tested whether PB2 M631L exerts a context-dependent effect when introduced into a polymerase already adapted to humans, as a means of assessing whether this mutation provides redundant or additive function relative to established mammalian-adaptive substitutions. To this end, we used the H1N1pdm09 virus, which emerged from swine in 2009 but carries polymerase genes of avian origin [13]. Human H1N1pdm09 viruses do not contain PB2 E627K or M631L but instead encode three adaptive substitutions—T271A, G590S, and Q591R—that enhance replication in human cells (Figure 3A; Table 1). Structural analysis shows that PB2 residues 590, 591, 627, and 631 cluster near the huANP32A interaction surface (Figure 3B). To test the effect of M631L in this context, we used a mini-genome assay with A/Hamburg/4/2009 and generated an isogenic “de-adapted” PB2 variant (271T/590A/591A) which had been shown previously to reduce polymerase activity [29,41] (Figure 3A). In both the wild-type and mutant backgrounds, we produced four PB2 variants encoding the combinations 627E/631M, 627E/631L, 627K/631M or 627K/631L.

Interestingly, the de-adapted 271T/590A/591A variant containing canonical avian-type 627E/631M showed an ~8-fold reduction in activity with huANP32A and ~10-fold with huANP32B compared to wild type (Figure 3C, left and middle panels, compare first gray with first blue bar). In contrast, this mutant retained full activity in the presence of the chicken ANP32A ortholog (Figure 3C, right panel). Remarkably, introducing M631L into PB2 significantly increased polymerase activity regardless of the signature 271/590/591 in the presence of either human ANP32 homolog. The fold changes in polymerase activity induced by PB2 M631L are indicated in the figure for each variant pair, showing that the strongest effects occur in the de-adapted context. Polymerase activity was increased to a similar extent when the canonical PB2 E627K was inserted instead (Figure 3C, left and middle panels). Introduction of PB2 M631L, PB2 E627K, or the 271T/590S/591R signature each increased polymerase activity relative to the de-adapted variant, whereas combinations of these mutations produced smaller additional effects. ANP32–polymerase interaction assays revealed distinct contributions of adaptive residues: only PB2 M631L increased binding to huANP32 proteins, whereas PB2 E627K and the 271T/590S/591R set had little effect (Figure 3D). This confirms the finding made in the 1918 polymerase that ANP32 binding levels measured by split-luciferase assays do not uniformly scale with polymerase output, most likely reflecting the contribution of both binding affinity and post-binding functional constraints to polymerase activity.

To determine the impact on virus replication, we generated recombinant H1N1pdm09 viruses expressing PB2 631M or 631L in either the wild-type or de-adapted background. The plaque assays on the MDCK-II cells showed pronounced phenotypes (Figure 4A): While the plaques of the wild-type virus were somewhat blurred and the cells were not always fully lysed, the corresponding 631L variant produced clear plaques of similar size. In the virus pair with the de-adapted signature, the differences were even more pronounced. While the de-adapted virus containing PB2 631M produced sharp, but small plaques, the single introduction of PB2 631L resulted in the formation of markedly larger plaques. Replication kinetics in the mammalian cell lines MDCK-II and Calu-3 cells confirmed these differences (Figure 4B). In the MDCK-II cells, PB2 M631L increased titres for both wild type (6.7-fold at 24 hpi) and de-adapted virus (443.8-fold at 24 hpi). In the Calu-3 cells, M631L significantly enhanced titres for the wild-type virus at 24 hpi, and at 48 hpi for the de-adapted variant. However, titres were lower, and fold changes were smaller compared to the more permissive MDCK-II cells.

Together, these findings show that PB2 M631L increases polymerase activity, ANP32 interaction, and replication in a human-adapted IAV background.

### 3.3. Testing Adaptation of Dairy Cow H5N1 Polymerase to Mammalian ANP32A Proteins via PB2 Positions 631 and 627

In an ongoing outbreak, avian H5N1 clade 2.3.4.4.b viruses have infected a considerable number of mammalian species, established circulation in dairy cattle in the United States and caused at least 72 human cases [44]. While avian precursor viruses carried the typical PB2 627E and 631M residues, most bovine and cattle-derived human viruses acquired the adaptive PB2 M631L mutation, whereas PB2 position 627 remained in the avian E configuration. Notably, one early human H5N1 case of this outbreak (A/Texas/37/2024) with 627K/631M likely came directly from birds [30] (Figure 5A). Having shown that PB2 M631L supports mammalian adaptation in the 1918 and 2009 strains, we next examined its effect in a recent H5N1 clade 2.3.4.4b polymerase. Polymerase activity and ANP32 binding were assessed with human and bovine ANP32A. Consistent with recent reports [34,35], PB2 M631L enhanced polymerase activity in the bovine H5N1 background. Here, these data are included to demonstrate conservation of ANP32-dependent effects across viral lineages rather than to assess pathogenicity or transmission. Using one of the first bovine H5N1 sequences, the wild-type polymerase showed 12.1- and 10.5-fold higher activity than a PB2 L631M revertant with human and bovine ANP32A, respectively (Figure 5B, left and middle panel). Activity for all the variants was high with chicken ANP32A, although M631L still produced a 6.1-fold increase. Similar effects were seen for PB2 E627K, though fold changes (4.9–6.3) were lower than for M631L. Again, the interaction assays showed that PB2 M631L, but not E627K, enhanced binding to mammalian ANP32A (Figure 5C, left and middle panels). As seen before, chicken ANP32A bound much more strongly, but the impact of polymerase variants was low (Figure 5C, right panel). Together, these experiments show that PB2 M631L increases polymerase activity and ANP32 binding in the bovine H5N1 background to an extent comparable to PB2 E627K, for human and bovine ANP32A.

## 4. Discussion

PB2 M631L has previously been identified in mammalian-adapted IAV originating in birds and has been proposed to contribute to polymerase adaptation [33,34]. The present study builds on these observations by providing a comparative analysis of PB2 M631L across multiple viral backgrounds, including historical pandemic and contemporary strains, and by linking its functional effects to ANP32-dependent host adaptation. The comparison with other adaptive changes in the polymerase revealed some degree of partial functional overlap despite different molecular mechanisms of action. Structural studies clarify how these mutations promote interaction with human ANP32 [25,26,45]. PB2 591R and 627K create a positively charged surface on the PB2 627 domain that binds the acidic LCAR region of ANP32A/B. PB2 D701N similarly removes a negative charge to support binding. Residue 631 is also part of the PB2 627 domain that contributes to the ANP32 interaction surface. Although the precise structural mechanism of PB2 M631L remains unresolved, its proximity suggests that it may subtly influence the interaction interface with ANP32 proteins. PB2 M631L does not alter charge, but we speculate that it may enhance ANP32A binding by interacting with the closely positioned K153 of ANP32 within the asymmetric polymerase dimer of the replicase form [26]. This idea can, however, currently only be considered a structure-guided hypothesis, and further structural or mutational analyses will be required to directly test this interaction. These diverse structural routes to human ANP32 compatibility highlight the dynamic interplay between viral polymerase and its essential host cofactor. This view is also supported by the finding that binding levels measured by split-luciferase assays do not uniformly scale with polymerase, indicating that polymerase activity reflects both binding affinity and post-binding functional constraints.

An important finding of our study is that each of the adaptive mutations studied here (591R/K, 627K, and 631L) can enhance polymerase activity with human ANP32 in the experimental systems used. Several observations are consistent with this interpretation: (i) In 1918 polymerases, M631L produced the largest activity gain in variants containing avian-type 627E rather than human-adapted 627K. (ii) In the 2009 H1N1pdm09 polymerase, M631L had a greater impact in the de-adapted version with suboptimal residues at positions 271, 590, and 591; consistent with this, H1N1pdm09 viruses have not acquired 627K or 701N during circulation, nor did these mutations alter virulence experimentally [46]. (iii) In vivo, H5N1 clade 2.3.4.4.b repeatedly crossed into mammals, with each spillover linked to a distinct single adaptive mutation. U.S. dairy cattle viruses carried only M631L [31], the first human isolate A/Texas/37/2024 and experimentally infected calves contained only 627K, and mink farm viruses in Spain expressed only 271A [47]. South American marine mammal isolates deviated slightly by carrying 591K, often combined with 701N [32]. Differences in ANP32A/B orthologs among mammalian species may explain the selection of different adaptive residues [48].

Importantly, the conclusions of this study are restricted to the experimental systems used, primarily polymerase activity and protein-interaction assays. The virus replication experiments were performed in a limited viral background using recombinant A/H1N1pdm09 viruses in cell culture. While these approaches provide mechanistic insight into PB2–ANP32 interactions, they do not directly assess viral adaptation or transmission in vivo. Therefore, broader implications regarding the range of influenza virus backgrounds in which PB2 M631L contributes to viral fitness, host adaptation, or epidemiological spread remain to be determined. Although the mutations discussed above have comparable effects on polymerase adaptation, their frequencies differ markedly among circulating IAV strains. Among all the human IAV isolates deposited in the NCBI Virus database before the 2009 H1N1pdm pandemic, PB2-627K appeared in 97.4% of the sequences and PB2-631M in 99.9%. In contrast, PB2-591K or PB2-591R were detected only at negligible frequencies during the same period. After the 2009 H1N1pdm virus replaced previously circulating H1N1 lineages, however, PB2-591R can be found in 40.2% of all the sequences. Notably, only H5N1 virus sequences exhibited a higher prevalence of the PB2-M631L mutation—13.5% of human and 6.5% of avian isolates (see Supplemental Table S2). Several factors may explain these distributional differences. Individual mutations may impose distinct fitness costs that influence the selection of specific polymerase variants. Mutational biases may also shape the observed patterns [49]. The PB2-E627K change can arise through a single transition mutation—G to A in the first codon position—via purine deamination, a process that occurs relatively frequently. In contrast, the PB2-M631L substitution requires a transversion (A to C) to convert AUG to CUG, a less probable event given the ~10:1 transition-to-transversion ratio [49]. Although the alternative leucine codon UUG could emerge through a simpler transition, it is used far less frequently in the human genome (0.13 versus 0.40 for CUG) [50], which may reduce the expression efficiency of the resulting polymerase. These mutational constraints and codon-usage considerations likely make acquisition of PB2-M631L considerably less frequent than PB2-E627K.

In conclusion, this study highlights that analysis of historical pandemic viruses can provide valuable context for interpreting adaptive features in contemporary influenza strains. The observed plasticity of IAV polymerase adaptation underscores the importance of continued surveillance and functional characterization of emerging variants.

## Figures and Tables

**Table 1:** Amino acid sequences of viruses and polymerases of this study indicating PB2 variants with a role in ANP32 interaction.

	PB2 Amino Acid Number					
Strain	271	590	591	627	631	Reference/Remarks
A/Brevig Mission/1/1918 (H1N1)	T	G	Q	K	M	1918 pandemic reference strain [5]
German 1918(H1N1) (MU-162)	T	G	Q	K	L	Novel sequence from German human lung sample [10]
A/Hamburg/4/2009 (H1N1pdm)	A	S	R	E	M	Human isolate of 2009 Swine flu pandemic [41]
Hamburg “de-adapted”	T	A	A	E	M	PB2 de-adapted
2024 H5N1cattle	T	G	Q	E	L	2024 US dairy cow sample [34,35,36]
A/mallard/Beijing/27-MA/2011 (H10N7)	T	G	Q	E	M/L	Avian virus with 631M acquired 631L during mouse passages [33]
A/chicken/Pakistan/UDL-01/2008 (H9N2)	T	G	Q	E	M/L	631L appeared during replication in ANP32A-modified chickens [42]

## Data Availability

The original contributions presented in this study are included in the article/Supplementary Material. Further inquiries can be directed to the corresponding author.

## Supplementary Materials

The following supporting information can be downloaded at https://www.mdpi.com/article/10.3390/microorganisms14040859/s1. Figure S1: PB2 alignment; Figure S2: H5N1 polymerase sequences; Table S1: Primers for mutagenesis and cloning; Table S2: Distribution of amino acid frequencies in PB2 from NCBI virus; File S1: Primary data and statistical analysis.

## Abbreviations

The following abbreviations are used in this manuscript:

ANP32	Acidic nuclear phosphoprotein of 32 kDa
PB1	Polymerase basic subunit 1
PB2	Polymerase basic subunit 2
PA	Polymerase acidic subunit
NP	nucleoprotein
