# Supplementary material for: Historical Pandemic and Contemporary Influenza A Viruses Reveal PB2 M631L as a Convergent Adaptation to Human ANP32
Source: Microorganisms. 2026 Apr 11;14(4):859. doi: 10.3390/microorganisms14040859 (PMC13118919; doi:10.3390/microorganisms14040859)
Supplement: Supplementary file 1 [file microorganisms-14-00859-s001.zip › Supplementary Figure S1 - PB2 alignment.pdf]

PB2 protein alignment

```
                                    10        20        30        40        50        60        70        80        90       100
Consensus                   MERIKELRDLMSQSRTREILTKTTVDHMAIIKKYTSGRQEKNPALRMKWMMAMKYPITADKRIMEMIPERNEQGQTLWSKTNDAGSDRVMVSPLAVTWWN
human consensus (1900-2008) ........N..................................S...................T................V.........I.........
avian PB2 consensus         ....................................................................................................
Brevig Mission 1918         ....................................................................................................
German 1918                 ...M................................................................................................
HH/04 2009pdm               .....................................................R..........D...................................
H5N1cattle 2024             .........................................................A..........................................

                                   110       120       130       140       150       160       170       180       190       200
Consensus                   RNGPTTSTVHYPKXYKTYFEKVERLKHGTFGPVHFRNQVKIRRRVDINPGHADLSAKEAQDVIMEVVFPNEVGARILTSESQLTITKEKKEELQDCKIXP
human consensus (1900-2008) ....VA..I.....................................................................................N.....
avian consensus             ....................................................................................................
Brevig Mission 1918         .......A............................................................................................
German 1918                 ....................................................................................................
HH/04 2009pdm               ..............................................T....................................A................
H5N1cattle 2024             ........I.............................I.............................................................

                                   210       220       230       240       250       260       270       280       290       300
Consensus                   LMVAYMLERELVRKTRFLPVAGGTSSVYIEVLHLTQGTCWEQMYTPGGEVRNDDVDQSLIIAARNIVRRATVSADPLASLLEMCHSTQIGGIRMVDILRQ
human consensus (1900-2008) ......................................................................A....................T........
avian consensus             ....................................................................................................
Brevig Mission 1918         ....................................................................................................
German 1918                 ....................................................................................................
HH/04 2009pdm               ........................G.............................................A....................V........
H5N1cattle 2024             ....................................................................................................

                                   310       320       330       340       350       360       370       380       390       400
Consensus                   NPTEEQAVDICKAAMGLRISSSFSFGGFTFKRTSGSSVKREEEVLTGNLQTLKIRVHEGYEEFTMVGRRATAILRKATRRLIQLIVSGRDEQSIAEAIIV
human consensus (1900-2008) .....................................................LT............K..........................V...V.
avian consensus             ....................................................................................................
Brevig Mission 1918         ....................................................................................................
German 1918                 ....................................................................................................
HH/04 2009pdm               ..............I........................K............................................................
H5N1cattle 2024             .............................................................G......................................

                                   410       420       430       440       450       460       470       480       490       500
Consensus                   AMVFSQEDCMIKAVRGDLNFVNRANQRLNPMHQLLRHFQKDAKVLFQNWGIEPIDNVMGMIGILPDMTPSTEMSXRGXRVSKMGVDEYSSTERVVVSIDR
human consensus (1900-2008) ..........V...................................L..........................................NA.........
avian consensus             ....................................................................................................
Brevig Mission 1918         ....................................................................................................
German 1918                 ....................................................................................................
HH/04 2009pdm               ....................................................S................N..............................
H5N1cattle 2024             ........................................N.....................................................I.....

                                   510       520       530       540       550       560       570       580       590       600
Consensus                   FLRVRDQRGNVLLSPEEVSETQGTEKLTITYSSSMMWEINGPESVLVNTYQWIIRNWETVKIQWSQBPTMLYNKMEFEPFQSLVPKAARGQYSGFVRTLF
human consensus (1900-2008) ..............................................I........................................I............
avian consensus             ....................................................................................................
Brevig Mission 1918         ......................................V.............................................................
German 1918                 ....................................................................................................
HH/04 2009pdm               .......................................................S..I............................T.SR.........
H5N1cattle 2024             ....................................................................................................

                                   610       620       630       640       650       660       670       680       690       700
Consensus                   QQMRDVLGTFDTVQIIKLLPFAAAPPXQSRMQFSSLTVNVRGSGMRILVRGNSPVFNYNKATKRLTVLGKDAGALTEDPDEGTAGVESAVLRGFLILGKE
human consensus (1900-2008) ............T...............................................T............T..........................
avian consensus             ....................................................................................................
Brevig Mission 1918         ....................................................................................................
German 1918                 ..............................L.....................................................................
HH/04 2009pdm               ............................................L......................................S................
H5N1cattle 2024             ..............................L.................I..........................A........................

                                   710       720       730       740       750       760
Consensus                   DXRYGPALSINELSNLAKGEKANVLIGQGDVVLVMKRKRDSSILTDSQTATKRIRMAIN-
human consensus (1900-2008) ...........................................................-
avian consensus             ...........................................................-
Brevig Mission 1918         ...........................................................-
German 1918                 ...........................................................-
HH/04 2009pdm               ...........................................................-
H5N1cattle 2024             ...........................................................-
```
