# Supplementary material for: Historical Pandemic and Contemporary Influenza A Viruses Reveal PB2 M631L as a Convergent Adaptation to Human ANP32
Source: Microorganisms. 2026 Apr 11;14(4):859. doi: 10.3390/microorganisms14040859 (PMC13118919; doi:10.3390/microorganisms14040859)
Supplement: Supplementary file 1 [file microorganisms-14-00859-s001.zip › Supplementary Figure S2 - H5N1 polymerase sequences.pdf]

Polymerase CDS of H5N1 clade 2.3.4.4.b sequences from US dairy cattle

Sequences were derived from: https://github.com/andersen-lab/avian-influenza

Coding Sequences

PB2

Source: Consensus_SRR28752653_PB2_cns_threshold_0.5_quality_20:
ATGGAGAGAATAAAAGAACTGAGAGATCTAATGTCACAGTCTCGCACTCGCGAGATACTAACCAAAACCACTG
TTGACCACATGGCCATAATCAAGAAGTACACATCAGGAAGACAAGAAAAGAACCCTGCACTCAGAATGAAAT
GGATGATGGCAATGAAATATCCAATCGCAGCAGACAAGCGAATAATGGAAATGATCCCTGAAAGGAATGAAC
AAGGACAAACCCTCTGGAGCAAGACAAATGATGCCGGATCAGATCGAGTGATGGTATCACCCCTGGCTGTGA
CATGGTGGAATAGGAATGGACCAACAACAAGTACAATTCACTATCCAAAGGTATACAAAACTTATTTTGAAAA
AGTTGAAAGGTTGAAACACGGGACCTTTGGCCCTGTACACTTCAGAAACCAAATTAAGATAAGACGGAGGGTC
GACATAAACCCGGGCCATGCTGACCTCAGCGCCAAAGAGGCGCAGGACGTAATCATGGAAGTTGTCTTTCCAA
ATGAAGTGGGAGCGAGAATACTGACGTCGGAATCACAATTGACAATAACAAAGGAAAAGAAAGAAGAACTCC
AGGACTGCAAAATCGCCCCTCTGATGGTTGCATACATGCTAGAAAGAGAGCTGGTCCGCAAGACAAGGTTCCT
CCCAGTTGCTGGTGGAACAAGCAGTGTCTACATTGAGGTGCTGCATTTGACCCAGGGAACATGCTGGGAGCA
GATGTATACTCCAGGAGGAGAAGTGAGAAACGATGATGTAGACCAGAGCTTGATCATTGCTGCCAGGAATAT
AGTAAGAAGAGCAACAGTGTCAGCAGACCCATTAGCATCTCTATTGGAGATGTGCCACAGCACACAAATTGGA
GGAATAAGGATGGTAGACATTCTTCGGCAAAATCCAACGGAGGAACAAGCCGTGGACATATGCAAGGCAGCA
ATGGGCTTGAGGATTAGCTCATCGTTCAGCTTTGGTGGATTCACTTTTAAAAGAACAAGTGGATCATCAGTCAA
AAGGGAAGAAGAGGTGCTTACGGGCAACCTTCAAACATTGAAAATAAGAGTACATGAGGGGTATGAAGGGTT
CACTATGGTTGGAAGAAGAGCAACGGCCATTCTCAGGAAAGCAACCAGAAGGCTGATTCAGCTAATAGTAAG
TGGAAGGGACGAACAGTCAATTGCTGAAGCAATAATCGTGGCCATGGTATTCTCACAAGAGGACTGCATGATA
AAGGCAGTCCGAGGTGATCTGAACTTTGTCAACAGGGCGAATCAGCGGCTGAATCCAATGCATCAGCTCTTGA
GACACTTCCAAAAGAATGCAAAAGTGCTTTTCCAAAATTGGGGAATTGAGCCCATTGACAATGTGATGGGAAT
GATCGGGATATTGCCTGACATGACTCCAAGTACTGAGATGTCACTGAGGGGAATAAGAGTCAGTAAGATGGG
AGTAGATGAATACTCCAGTACAGAGCGGGTAATAGTAAGCATCGACCGATTTTTAAGAGTTCGAGACCAACGG
GGGAACGTACTACTGTCACCCGAAGAAGTCAGCGAGACACAAGGGACAGAGAAATTGACAATCACTTATTCG
TCATCAATGATGTGGGAGATCAATGGTCCTGAGTCGGTGTTGGTCAATACTTATCAGTGGATAATCAGAAACT
GGGAAACAGTAAAAATTCAATGGTCACAAGATCCCACAATGTTGTATAATAAGATGGAGTTCGAGCCATTCCA
GTCTCTGGTCCCTAAGGCAGCCAGGGGTCAATACAGTGGGTTCGTGAGGACACTATTTCAGCAAATGCGAGAT
GTGCTTGGAACATTTGACACTGTTCAGATAATAAAACTTCTCCCCTTTGCTGCTGCCCCACCGGAACAAAGTAG
ACTGCAATTCTCCTCTCTGACTGTGAATGTGAGAGGATCAGGAATGAGAATACTGATAAGAGGCAATTCTCCA
GTGTTCAATTACAACAAGGCCACCAAGAGGCTCACAGTTCTCGGGAAAGATGCAGGTGCATTGGCCGAAGATC
CAGATGAAGGCACAGCTGGAGTGGAGTCTGCTGTTTTAAGAGGATTCCTCATTTTGGGCAAAGAAGACAAGA
GATATGGCCCAGCACTGAGCATCAATGAGCTGAGCAATTTGGCAAAGGGAGAGAAGGCTAATGTGCTAATTG
GGCAAGGAGACGTGGTGTTGGTGATGAAACGGAAACGGGACTCTAGCATACTTACTGACAGCCAGACAGCGA
CCAAAAGAATTCGGATGGCCATCAATTAG

PB1

Source: Consensus_SRR28752562_PB1_cns_threshold_0.5_quality_20

ATGGATGTCAATCCGACCTTACTCTTCTTGAAAGTTCCAGCGCAAAATGCCATAAGCACCACATTCCCGTATACT
GGAGATCCTCCATACAGCCATGGAACAGGAACAGGATATACCATGGACACAGTTAACAGAACACATCAATATT
CAGAAAAAGGGAAATGGACAACAAACTCAGAAACCGGGGCACCTCAACTCAATCCAATTGATGGACCATTGC
CTGATGACAATGAGCCAAGTGGATATGCACAAACGGACTGCGTCCTTGAAGCAATGGCTTTCCTTGAAGAATC
CCATCCAGGAATCTTTGAAAACTCGTGTCTTGAAACGATGGAAGTTGTTCAACAAACAAGAGTGGACAAGTTG
ACCCAAGGCCGTCAGACTTATGATTGGACATTAAACAGAAATCAGCCGGCTGCAACTGCATTAGCTAATACTAT
AGAGGTCTTCAGATCGAACGGTCTTACAGCTAATGAATCAGGAAGGCTAATAGATTTCCTCAAGGATGTGGTG
GAATCAATGGATAAAGAGGAAATAGAAATAACAACGCATTTCCAAAGGAAAAGAAGAGTGAGAGACAACAT
GACCAAGAAAATGGTCACACAACGGACGATAGGAAAGAAGAAACAAAGGTTAAACAAAAGGAGCTATCTGAT
AAGAGCATTGACACTGAACACAATGACAAAAGACGCCGAAAGAGGCAAATTAAAGAGAAGGGCAATTGCAAC
ACCCGGAATGCAAATCAGAGGGTTTGTGTACTTTGTTGAAACATTAGCAAGGAGCATTTGTGAGAAACTTGAA
CAATCTGGACTCCCAGTTGGAGGCAATGAAAAGAAGGCCAAACTAGCAAATGTTGTGAGAAAGATGATGACT
AATTCGCAAGACACAGAGCTCTCTTTCACAATCACGGGAGACAACACTAAATGGAATGAGAACCAGAATCCTA
GGATGTTTCTGGCAATGATAACATATATAACAAGGAACCAACCTGAATGGTTCAGGAATGTATTGAGCATTGC
ACCTATAATGTTCTCAAACAAAATGGCAAGACTAGGGAAAGGATACATGTTCGAAAGTAAGAGCATGAAGCTT
CGAACACAAATACCGGCAGAAATGCTAGCGAGCATTGATCTGAAATACTTCAATGAGTCAACAAGGAAGAAA
ATAGAGAAGATAAGACCTCTTCTAATAGATGGTACGGCCTCATTAAGCCCTGGAATGATGATGGGCATGTTCA
ACATGCTGAGTACAGTTCTGGGAGTTTCGATTCTAAATCTAGGGCAAAAGAAGTACACCAAAACAACATACTG
GTGGGATGGACTACAATCTTCTGATGACTTTGCTCTCATCGTGAATGCTCCAAATCATGAGGGAATACAAGCA
GGAGTAGACAGATTCTATAGAACCTGCAAGCTGGTAGGAATCAATATGAGCAAAAAGAAGTCATACATAAAC
AGGACAGGAACATTTGAATTCACAAGTTTTTTCTATCGCTATGGATTTGTAGCCAATTTCAGCATGGAGTTGCC
CAGCTTTGGAGTTTCTGGGATCAATGAATCTGCAGACATGAGCATTGGAGTAACAGTGATAAAGAACAACATG
ATCAACAATGATCTTGGACCAGCAACAGCCCAAATGGCTCTACAGCTATTCATCAAGGATTACAGATACACATA
TCGATGTCACAGAGGAGACACACAAATTCAAACAAGGAGGTCATTCGAGCTGAAAAAGTTATGGGAACAAAC
CCGCTCAAAACCAGGACTGCTGGTCTCAGATGGAGGGCCAAATCTATACAATATCCGAAATCTCCACATTCCGG
AAGTCTGCTTAAAATGGGAGCTAATGGACGAAGACTATCAGGGAAGGCTTTGTAATCCCCTGAATCCGTTTGT
AAGCCACAAAGAAATAGAGTCTGTGAACAATGCTGTGGTGATGCCAGCTCATGGCCCAGCTAAGAGTATGGA
ATATGATGCTGTTGCCACCACTCACTCCTGGATCCCTAAAAGGAACCGCTCTATTCTTAATACAAGCCAAAGGG
GAATCCTTGAAGACGAACAGATGTATCAAAAGTGCTGCAATCTATTTGAAAAATTCTTCCCTAGCAGTTCATAC
AGGAGGCCGGTTGGAATTTCCAGCATGGTGGAGGCCATGGTTTCTAGGGCCCGAATTGATGCACGAATTGAC
TTCGAATCTGGACGGATTAAGAAGGAGGAGTTTGCTGAGATCATGAAGATCTGTTCCACCATTGAAGAGCTCA
GACGGCAGAAATAG

PA:

Consensus_SRR28752656_PA_cns_threshold_0.5_quality_20

ATGGAAGACTTTGTGCGACAATGCTTCAATCCAATGATTGTCGAGCTTGCGGAAAAAGCAATGAAAGAATATG
GGGAAGATCCGAAAATCGAGACAAACAAATTTGCCGCAATATGCACACACTTAGAAGTCTGTTTCATGTATTC
GGATTTCCATTTTATTGACGAACGAGGCGAATCAATGATTGTAGAATCTGGTGATCCAAATGCATTATTGAAAC
ACCGATTTGAGATAATCGAAGGGAGAGACCGAGCAATGGCCTGGACAGTGGTGAATAGTATCTGCAACACCA
CAGGGGTCGAAAAGCCCAAATTCCTCCCTGATTTGTATGACTACAGAGAGAACAGATTCATTGAAATTGGAGT
AACGCGAAGGGAAGTTCACATATACTATTTGGAAAAAGCCAACAAGATAAAATCAGAGAAAACACATATTCAC
ATATTCTCATTCACTGGAGAGGAAATGGCCACCAAGGCGGACTACACCCTTGATGAAGAGAGCAGAGCAAGA
ATAAAAACCAGACTGTTCACTATAAGACAAGAAATGGCCAGTAGAGGTCTATGGGATTCCTTTCGTCAATCCG
AGAGAGGCGAAGAGACAATTGAAGAAAGATTTGAAATCACAGGAACCATGCGCAGGCTTGCCGACCAAAGTA
TCCCACCGAACTTCTCCAGCCTTGAAAACTTTAGAGCCTATGTGGATGGATTCGAACCGAACGGCTGCATTGAG

GGCAAGCTTTCTCAAATGTCAAAAGAGGTGAACGCCAGAATTGAGCCATTTCTGAAGACAACACCACGCCCTC
TCAGATTACCTGATGGGCCTCCCTGTCCTCAGCGGTCGAAGTTCTTGCTGATGGATGCCCTTAAGTTGAGCATC
GAAGACCCTAGTCATGAGGGGGAGGGCATACCGCTGTATGATGCAATCAAATGCATGAAGACATTTTTTGGCT
GGAAAGAGCCCAACATCGTAAAGCCGCATGAGAAAGGCATAAACCCTAATTACCTCCTGGCTTGGAAGCAGG
TGCTGGCAGAACTTCAAGACATTGAAAATGAGGAGAAAATTCCAAAAACAAAGAACATGAAGAAAACAAGCC
AATTGAAGTGGGCACTTGGTGAGAACATGGCTCCAGAAAAAGTGGACTTTGAGGACTGCAAAGATGTTAGCG
ATCTAAGACAGTACGACAGTGACGAACCAGAGTCTAGATCACTAGCAAGCTGGATTCAGAGTGAATTCAACAA
GGCATGCGAACTGACAGATTCGAGTTGGATTGAACTTGATGAGATAGGGGAAGACGTTGCTCCAATCGAACA
CATTGCGAGTGTGAGGAGGAACTATTTCACAGCGGAGGTATCCCATTGCAGGGCCACTGAATACATAATGAA
GGGAGTATACATAAACACAGCCCTATTGAATGCATCCTGTGCAGCCATGGATGACTTCCAATTGATTCCAATGA
TAAGTAAGTGCAGAACTAAAGAAGGAAGACGGAGGACAAATCTGTATGGATTCATTATAAAAGGAAGATCCC
ATTTGAGGAATGACACCGATGTGGTAAACTTTGTGAGCATGGAATTCTCTCTAACTGACCCGAGGCTAGAGCC
ACACAAATGGGAAAAGTACTGTGTTCTTGAGATAGGAGACATGCTCCTACGGACTGCAATAGGCCAAGTATTG
AGGCCCATGTTCCTGTATGTGAGAACCAATGGAACTTCCAAGATCAAAATGAAATGGGGCATGGAGATGAGG
CGATGCCTTCTTCAGTCCCTTCAACAAATTGAGAGCATGATTGAGGCCGAATCTTCTGTCAAAGAGAAGGACAT
GTCCAAGGAATTCTTTGAAAACAAATCAGAAACATGGCCAATTGGAGAATCACCCAAAGGGGTGGAGGAAGG
CTCTATTGGGAAAGTATGCAGAACATTGCTAGCAAAGTCTGTGTTCAACAGCCTATATGCATCTCCTCAACTCG
AGGGGTTTTCAGCTGAATCAAGAAAATTGCTTCTCATTGTTCAGGCACTTAGGGACAACCTGGAACCTGGAAC
CTTCGATCTTGGGGGGCTATATGAAGCAATTGAGGAGTGCCTGATTAACGATCCCTGGGTTTTGCTTAATGCAT
CTTGGTTCAACTCCTTCCTCACACATGCACTGAAATAG

NP:

Consensus_SRR28752652_NP_cns_threshold_0.5_quality_20

ATGGCGTCTCAAGGCACCAAACGATCCTATGAACAAATGGAAACTGGTGGGGAACGCCAGAATGCCACTGAA
ATCAGAGCATCTGTTGGAAGAATGGTTGGCGGAATCGGGAGATTCTACATACAGATGTGCACTGAGCTCAAAC
TCAGTGATCACGAAGGGAGGCTGATCCAAAACAGCATAACCATAGAAAGGATGGTTCTCTCGGCATTTGATGA
AAGGAGGAACAAGTATCTGGAGGAACATCCCAGTGCTGGAAAGGATCCCAAGAAGACTGGAGGTCCAATCTA
CAGGAGGAGAGATGGCAAATGGATGAGAGAGTTGATCCTCTACGACAAAGAAGAGATCAGAAGAATTTGGC
GTCAAGCTAATAATGGAGAGGATGCAACTGCTGGTCTCACTCACTTGATGATTTGGCATTCCAATCTGAATGAT
GCCACATACCAGAGAACAAGGGCACTTGTGCGTACTGGAATGGATCCTAGGATGTGCTCACTGATGCAAGGCT
CAACCCTCCCTAGGAGATCCGGGGCTGCTGGAGCGGCAGTGAAAGGAGTTGGAACAATGGTGATGGAATTGA
TTCGAATGATCAAACGAGGAATCAATGATCGGAATTTCTGGAGAGGTGAAAACGGACGGAGAACCAGGATTG
CCTACGAGAGAATGTGCAACATCCTCAAGGGAAAGTTCCAAACAGCAGCACAACGAGCAATGATGGACCAAG
TGAGGGAAAGCCGGAATCCTGGGAATGCTGAAATTGAAGATCTCATCTTTCTCGCACGATCTGCTCTCATCCTG
AGGGGATCAGTGGCTCATAAGTCCTGTCTGCCTGCTTGCGTGTATGGACTTGCTGTAGCCAGTGGATATGACT
TTGAAAGAGAGGGATACTCTCTAGTCGGAATTGATCCTTTCCGTCTGCTCCAGAACAGTCAAGTTTTCAGTCTC
ATCAGACCGAATGAAAATCCAGCTCACAAAAGTCAGCTGGTATGGATGGCATGCCACTCTGCAGCATTTGAGG
ATCTGAGAGTGTCAAGCTTCATCAGAGGAACAAGAGTAGTCCCAAGAGGACAACTGTCCACCAGAGGAGTTC
AGATTGCTTCAAATGAAAACATGGAGACAATGGATTCCAGTACTCTTGAACTGAGGAGCAGATACTGGGCTAT
AAGAACAAGAAGTGGAGGAAACACCAACCAACAGAGAGCATCTGCAGGACAAATCAGCGTACAGCCCACATT
CTCTGTGCAGAGAAACCTCCCATTCGAGAGAGCAACCATCATGGCAGCATTTACGGGAAACACTGAAGGCAGA
ACTTCAGACATGAGAACTGAGATCATAAGGATGATGGAAAATGCCAGACCTGAAGATGTGTCTTTCCAGGGGC
GGGGAGTCTTCGAGCTCTCGGACGAAAAGGCAACGAACCCGATCGTGCCTTCCTTTGACATGAACAATGAAG
GATCTTATTTCTTCGGAGACAATGCAGAGGAGTATGACAATTAA

Protein sequences

PB2:

Source: Consensus_SRR28752652_PB2_cns_threshold_0.5_quality_20 translation:
MERIKELRDLMSQSRTREILTKTTVDHMAIIKKYTSGRQEKNPALRMKWMMAMKYPIAADKRIMEMIPERNEQG
QTLWSKTNDAGSDRVMVSPLAVTWWNRNGPTTSTIHYPKVYKTYFEKVERLKHGTFGPVHFRNQIKIRRRVDINP
GHADLSAKEAQDVIMEVVFPNEVGARILTSESQLTITKEKKEELQDCKIAPLMVAYMLERELVRKTRFLPVAGGTSSV
YIEVLHLTQGTCWEQMYTPGGEVRNDDVDQSLIIAARNIVRRATVSADPLASLLEMCHSTQIGGIRMIDILRQNPTE
EQAVDICKAAMGLRISSSFSFGGFTFKRTSGSSVKREEEVLTGNLQTLKIRVHEGYEGFTMVGRRATAILRKATRRLIQ
LIVSGRDEQSIAEAIIVAMVFSQEDCMIKAVRGDLNFVNRANQRLNPMHQLLRHFQKNAKVLFQNWGIEPIDNVM
GMIGILPDMTPSTEMSLRGIRVSKMGVDEYSSTERVIVSIDRFLRVRDQRGNVLLSPEEVSETQGTEKLTITYSSSMM
WEINGPESVLVNTYQWIIRNWETVKIQWSQDPTMLYNKMEFEPFQSLVPKAARGQYSGFVRTLFQQMRDVLGTF
DTVQIIKLLPFAAAPPEQSRLQFSSLTVNVRGSGMRILIRGNSPVFNYNKATKRLTVLGKDAGALAEDPDEGTAGVES
AVLRGFLILGKEDKRYGPALSINELSNLAKGEKANVLIGQGDVVLVMKRKRDSSILTDSQTATKRIRMAIN

PB1:

Source: Consensus_SRR28752652_PB1_cns_threshold_0.5_quality_20 translation:
MDVNPTLLFLKVPAQNAISTTFPYTGDPPYSHGTGTGYTMDTVNRTHQYSEKGKWTTNSETGAPQLNPIDGPLPD
DNEPSGYAQTDCVLEAMAFLEESHPGIFENSCLETMEVVQQTRVDKLTQGRQTYDWTLNRNQPAATALANTIEVF
RSNGLTANESGRLIDFLKDVVESMDKEEIEITTHFQRKRRVRDNMTKKMVTQRTIGKKKQRLNKRSYLIRALTLNTM
TKDAERGKLKRRAIATPGMQIRGFVYFVETLARSICEKLEQSGLPVGGNEKKAKLANVVRKMMTNSQDTELSFTITG
DNTKWNENQNPRMFLAMITYITRNQPEWFRNVLSIAPIMFSNKMARLGKGYMFESKSMKLRTQIPAEMLASIDLK
YFNESTRKKIEKIRPLLIDGTASLSPGMMMGMFNMLSTVLGVSILNLGQKKYTKTTYWWDGLQSSDDFALIVNAPN
HEGIQAGVDRFYRTCKLVGINMSKKKSYINRTGTFEFTSFFYRYGFVANFSMELPSFGVSGINESADMSIGVTVIKNN
MINNDLGPATAQMALQLFIKDYRYTYRCHRGDTQIQTRRSFELKKLWEQTRSKPGLLVSDGGPNLYNIRNLHIPEVC
LKWELMDEDYQGRLCNPLNPFVSHKEIESVNNAVVMPAHGPAKSMEYDAVATTHSWIPKRNRSILNTSQRGILED
EQMYQKCCNLFEKFFPSSSYRRPVGISSMVEAMVSRARIDARIDFESGRIKKEEFAEIMKICSTIEELRRQK

PA:

Source: Consensus_SRR28752652_PA_cns_threshold_0.5_quality_20 translation:
MEDFVRQCFNPMIVELAEKAMKEYGEDPKIETNKFAAICTHLEVCFMYSDFHFIDERGESMIVESGDPNALLKHRFE
IIEGRDRAMAWTVVNSICNTTGVEKPKFLPDLYDYRENRFIEIGVTRREVHIYYLEKANKIKSEKTHIHIFSFTGEEMAT
KADYTLDEESRARIKTRLFTIRQEMASRGLWDSFRQSERGEETIEERFEITGTMRRLADQSIPPNFSSLENFRAYVDGF
EPNGCIEGKLSQMSKEVNARIEPFLKTTPRPLRLPDGPPCPQRSKFLLMDALKLSIEDPSHEGEGIPLYDAIKCMKTFF
GWKEPNIVKPHEKGINPNYLLAWKQVLAELQDIENEEKIPKTKNMKKTSQLKWALGENMAPEKVDFEDCKDVSDL
RQYDSDEPESRSLASWIQSEFNKACELTDSSWIELDEIGEDVAPIEHIASVRRNYFTAEVSHCRATEYIMKGVYINTAL
LNASCAAMDDFQLIPMISKCRTKEGRRRTNLYGFIIKGRSHLRNDTDVVNFVSMEFSLTDPRLEPHKWEKYCVLEIG
DMLLRTAIGQVLRPMFLYVRTNGTSKIKMKWGMEMRRCLLQSLQQIESMIEAESSVKEKDMSKEFFENKSETWPI
GESPKGVEEGSIGKVCRTLLAKSVFNSLYASPQLEGFSAESRKLLLIVQALRDNLEPGTFDLGGLYEAIEECLINDPWVL
LNASWFNSFLTHALK

NP:

Source: Consensus_SRR28752652_NP_cns_threshold_0.5_quality_20 translation:
MASQGTKRSYEQMETGGERQNATEIRASVGRMVGGIGRFYIQMCTELKLSDHEGRLIQNSITIERMVLSAFDERRN
KYLEEHPSAGKDPKKTGGPIYRRRDGKWMRELILYDKEEIRRIWRQANNGEDATAGLTHLMIWHSNLNDATYQRT

RALVRTGMDPRMCSLMQGSTLPRRSGAAGAAVKGVGTMVMELIRMIKRGINDRNFWRGENGRRTRIAYERMC
NILKGKFQTAAQRAMMDQVRESRNPGNAEIEDLIFLARSALILRGSVAHKSCLPACVYGLAVASGYDFEREGYSLVG
IDPFRLLQNSQVFSLIRPNENPAHKSQLVWMACHSAAFEDLRVSSFIRGTRVVPRGQLSTRGVQIASNENMETMDS
STLELRSRYWAIRTRSGGNTNQQRASAGQISVQPTFSVQRNLPFERATIMAAFTGNTEGRTSDMRTEIIRMMENA
RPEDVSFQGRGVFELSDEKATNPIVPSFDMNNEGSYFFGDNAEEYDN
