# Supplementary material for: Historical Pandemic and Contemporary Influenza A Viruses Reveal PB2 M631L as a Convergent Adaptation to Human ANP32
Source: Microorganisms. 2026 Apr 11;14(4):859. doi: 10.3390/microorganisms14040859 (PMC13118919; doi:10.3390/microorganisms14040859)
Supplement: Supplementary file 1 [file microorganisms-14-00859-s001.zip › Supplementary Table S1 - Primers.pdf]

Supplemental Table S1: Primers for mutagenesis and cloning

Name	Sequence	remarks
BM/MU K627E f	CAGCCGCTCCACCAGAGCAAAGTAGAATGC	Introduces K627E for BM wt or MU/L631M as template
BM/MU K627E r	GCATTCTACTTTGCTCTGGTGGAGCGGCTG	Introduces K627E for BM wt or MU/L631M as template
BM M631L f	CCACCAAAGCAAAGTAGATTGCAGTTCTCCTC TCTG	Introduces M631L for BM
BM M631L r	CAGAGAGGAGAACTGCAATCTACTTTGCTTTG GTGG	Introduces M631L for BM
BM/MU K627E/M631L f	CAGCCGCTCCACCAGAGCAAAGTAGATTGC	Introduces K627E for BM/M631L or MU wt as template
BM/MU K627E/M631L r	GCAATCTACTTTGCTCTGGTGGAGCGGCTG	Introduces K627E for BM M631L or MU-162 wt as template)
BM, MU PB1 pCI f	AAAACTCGAGACCATGGATGTCAATCCGACTT TACTTTTC	For PB1 subcloning into pCI-LL-Gaussia luc2
BM, MU PB1 pCI r	AAAAGCGGCCGCCTTTTGCCGTCTGAGCTCTT C	For PB1 subcloning into pCI-LL-Gaussia luc2
MU L631M f	CCACCAAAGCAAAGTAGAATGCAGTTCTCCTC TCTG	Introduces L631M for MU wt
MU L631M r	CAGAGAGGAGAACTGCATTCTACTTTGCTTTG GTGG	Introduces L631M for MU wt
HH A271T f	CATAGTAAGAAGAGCAACAGTGTCAGCAGAC CC	Introduces A271T for HH wt
HH A271T r	GGGTCTGCTGACACTGTTGCTCTTCTTACTATG	Introduces A271T for HH wt
HH SR590AA f	GTCCCTAAGGCAACCAGAGCCGCGTACAGTGG ATTCGTAAG	Introduces SR590AA for HH wt
HH SR590AA r	GTCCCTAAGGCAACCAGAGCCGCGTACAGTGG ATTCGTAAG	Introduces SR590AA for HH wt

HH04 E627K f	GCTGCTGCTCCACCAAAACAGAGTAGGATGC	Introduces E627K for HH wt
HH04 E627K r	GCATCCTACTCTGTTTTGGTGGAGCAGCAGC	Introduces E627K for HH wt
HH04 M631L f	CCACCAGAACAGAGTAGGCTGCAATTTTCCTC ATTGAC	Introduces M631L for HH wt
HH04 M631L r	GTCAATGAGGAAAATTGCAGCCTACTCTGTTC TGGTGG	Introduces M631L for HH wt
HH04 E627K /M631L f	GCTGCTGCTCCACCAAAACAGAGTAGGCTGC	Introduces E627K for HH M631L
HH04 E627K /M631L r	GCAGCCTACTCTGTTTTGGTGGAGCAGCAGC	Introduces E627K for HH M631L
HH PB2 f	AAAAGGTCTCAGGGAGCCGCCACCATGGAGA GAATAAAAGAACTGAGAG	For subcloning into pCAGGS
HH PB2 r	AAAAGGTCTCCTATTACTAATTGATGGCCATC CGAATTC	For subcloning into pCAGGS
HH PB1 f	AAAAGGTCTCAGGGAGCCGCCACCATGGATGT CAATCCGACTCTACTTTTC	For subcloning into pCAGGS
HH PB1 r	AAAAGGTCTCCTATTTCATTATTTTTGCCGTCT GAGT	For subcloning into pCAGGS
HH PA f	AAAAGGTCTCAGGGAGCCGCCACCATGGAAG ACTTTGTGCGACAATG	For subcloning into pCAGGS
HH PA r	AAAAGGTCTCCTATTCTACTTCAGTGCATGTGT GAGG	For subcloning into pCAGGS
HH NP f		
HH NP r	CAATGCAGAGGAGTATGACAATTAAAATAGG AGACCTTTT	For subcloning into pCAGGS
HH PB1 pcI f	AAAACTCGAGACCATGGATGTCAATCCGACTC TACTTTTC	For subcloning into pCI to generate Luciferase fusion proteins
HH PB1 pcI r	AAAAGCGGCCGCTTTTTGCCGTCTGAGTTCTTC	For subcloning into into pCI-LL-Gaussia luc2
H5 PB2 f	AAAA*GGTCTC*AGGGAGCCGCCACCATGGAGA GAATAAAGGAATTAAGAG	For subcloning into pCAGGS

H5 PB2 r	AAAAGGTCTCCTATTACTAATTGATGGCCATC CGAATTC	For subcloning into pCAGGS
H5 PB1 f	AAAAGGTCTCAGGGAGCCGCCACCATGGATGT CAATCCGACTTTACTTTTC	For subcloning into pCAGGS
H5 PB1 r	AAAAGGTCTCCTATTACTAATGGATGTCAATC CGACTTTAC	For subcloning into pCAGGS
H5 PA r	AAAAGGTCTCAGGGAGCCGCCACCATGGAAG ATTTTGTGCGACAATG	For subcloning into pCAGGS
H5 PA r	AAAAGGTCTCCTATTCTATTTCAGTGCATGTGC GAGG	For subcloning into pCAGGS
H5 NP f	AAAAGGTCTCAGGGAGCCGCCACCATGGCGTC TCAAGGCACCAAACG	For subcloning into pCAGGS
H5 NP r	AAAAGGTCTCCTATTTTAATTGTCATACTCCTC TGCATTG	For subcloning into pCAGGS
H5 PB1 pCI f	AAAACTCGAGACCATGGATGTCAATCCGACCT TACTC	For PB1 subcloning into pCI-LL-Gaussia luc2
H5 PB1 pCI r	AAAAGCGGCCGCTTTCTGCCGTCTGAGCTCTT C	For PB1 subcloning into pCI-LL-Gaussia luc2
bovANP32 pCI f	TAGCCTCGAGACCATGGACATGGACAAACGG ATTC	For PB1 subcloning into pCI-LL-Gaussia luc1
bovANP32 pCI r	GCGGCCGCCTTATCGTCGTCATCCTTGTAATC	For PB1 subcloning into pCI-LL-Gaussia luc1
MBT Uni12	ACGCGTGATCAGCAAAAGCAGG	Multisegment PCR for virus sequencing
MBT Uni12-G	ACGCGTGATCAGCGAAAGCAGG	Multisegment PCR for virus sequencing
MBT Uni13	ACGCGTGATCAGTAGAAACAAGG	Multisegment PCR for virus sequencing
