# Supplementary material for: Historical Pandemic and Contemporary Influenza A Viruses Reveal PB2 M631L as a Convergent Adaptation to Human ANP32
Source: Microorganisms. 2026 Apr 11;14(4):859. doi: 10.3390/microorganisms14040859 (PMC13118919; doi:10.3390/microorganisms14040859)
Supplement: Supplementary file 1 [file microorganisms-14-00859-s001.zip › Supplementary Table S2 - PB2 amino acid frequencies.pdf]

Distribution of amino acid frequencies in PB2 from NCBI virus

(https://www.ncbi.nlm.nih.gov/labs/virus/vssi/#/)

Downloaded on 2025/01/12. Shown are all frequencies above 1%

		Amino Acid					
	Sequences	271	590	591	627	631	701
1900-2008, all subtypes							
Human	3.969	96.4% A<br>3.6% T	72.0% G<br>27.9% S	99.9% G	97.4% K<br>2.5% E	99.9% M	99.4% D
Birds	5.689	98.9% T	87.8% G<br>11.6% S	99.1% Q	96.2% E<br>3.1%K	99.8% M	99.9% D
2009-today, all subtypes							
Human	54.453	99.5% A	98.4% S<br>1.2% G	59.7% Q<br>40.2% R	59.4% K<br>40.5% E	99.9% M	99.9% D
Birds	2.599	99.8% A	95.4% G<br>4.2% S	99.4% Q	98.4% E	97.7% M<br>2.1% L	99.8% D
1900- today, H5N1							
human	252	85.7% T<br>6.7% L<br>2.4% A<br>1.6% V<br>1.2% M	98.0% G<br>1.2% S	98.4% Q<br>1.2% K	73.8% E<br>26.2% K	86.1% M<br>13.5% L	95.6% D<br>4.4% N
birds	7.315	95.5% T	94.8% G	97.3% Q	92.7% E<br>4.3% K	90.3% M<br>6.5% L	96.8% D<br>1.1% S
cattle	1.813	100% T	99.9% G	99.2% Q	99.9% E	100% L	100% D
